# Injury and recovery patterns of the blood-testis barrier and spermatogenic function in a surgical unilateral cryptorchidism model in rats

**DOI:** 10.1186/s12958-026-01590-5

**Published:** 2026-07-16

**Authors:** Yan He, Run-Long Wang, Li-Peng Li, Hao-Xuan Yang, Yi-Fei Liu, Wen-Nuo Zhang, Hao-Yang Song, Ming-Xin Zhao, Jin-Chun Qi

**Affiliations:** 1 Urology Department, The Second Hospital of Hebei Medical University, Shijiazhuang, Hebei 050000 China; 2 Reproductive Medicine Department, The Second Hospital of Hebei Medical University, Shijiazhuang, Hebei 050000 China; 3 Hebei Medical University, Shijiazhuang, Hebei 050000 China

**Keywords:** Cryptorchidism, Rat, Blood-Testis Barrier, Spermatogenesis

## Abstract

**Background:**

Cryptorchidism is one of the most common congenital disorders of the pediatric urogenital system. In addition to regulating the passage of substances into and out of the seminiferous epithelium, the blood-testis barrier (BTB) plays a critical role in maintaining a stable microenvironment for spermatogenesis. BTB alterations in cryptorchidism remain poorly characterized. This study aimed to establish a surgical unilateral cryptorchidism model in rats (SUCMR) and to investigate the injury and recovery patterns of both the BTB and spermatogenic function within this model.

**Methods:**

The SUCMR was performed on 6-week-old male Sprague-Dawley rats, and testicular changes were observed after maintaining the modeling state for multiple time points. Simultaneously, another batch of rats underwent testicular descent surgery after maintaining the modeling for different durations to observe testicular recovery. The testis mass and volume, epididymal mass, epididymal sperm count and sperm malformation rate were quantified. After HE staining of testicular tissue, the seminiferous tubule structure was observed, and the seminiferous tubule diameter and seminiferous epithelium thickness were statistically analyzed. Biotin tracing experiments and transmission electron microscopy were performed to detect the BTB integrity. The expression of key BTB proteins ZO-1, OCCLUDIN, and CLAUDIN-11 was detected by WB. According to the normality of data distribution, a paired t-test or the Wilcoxon signed-rank test, an unpaired t-test or the Mann-Whitney U test, a one-way analysis of variance (ANOVA) or the Kruskal-Wallis test were used. A p-value of less than 0.05 was considered statistically significant.

**Results:**

In SUCMR, sloughing of the seminiferous epithelium was observed, resulting in testicular atrophy and spermatogenic abnormalities. The SUCMR impaired the integrity of the BTB and leaded to a progressive decrease in the expression of the key barrier proteins. Testicular atrophy and spermatogenic function injury induced by short-term SUCMR were reversible. But the disruption to the BTB was irreversible when the SUCMR was maintained for 4 weeks or longer.

**Conclusion:**

In this model, spermatogenic dysfunction and BTB injury progressed with increasing duration of abdominal retention, and abnormalities induced by 4 or 6 weeks of SUCMR did not recover within the post-orchiopexy observation period.

**Supplementary Information:**

The online version contains supplementary material available at https://doi.org/10.1186/s12958-026-01590-5.

## Background

Cryptorchidism is one of the most common congenital disorders of the pediatric urogenital system [1]. It can impair spermatogenic function, leading to infertility in adulthood [2]. Notably, spermatogenic function in some patients fails to recover even after successful orchiopexy [3]. The precise pathogenesis of cryptorchidism remains incompletely understood [4], warranting further in-depth investigation.

Animal models that simulate clinical pathological states are indispensable for biomedical research, providing critical data for drug development and therapeutic strategies. The availability of a stable and reliable animal model is therefore fundamental to the study of any disease. For cryptorchidism, where human cryptorchid testicular tissue is difficult to obtain and its collection raises ethical concerns, animal experiments serve as a vital alternative for mechanistic exploration. Currently, surgical induction is the primary method for establishing cryptorchidism models [5], although other approaches, such as pharmacological induction during pregnancy, also exist [6]. However, most existing studies have focused on modeling at a single time point [7], and a systematic, comprehensive evaluation of the surgical cryptorchidism model over time is still lacking.

The blood-testis barrier (BTB) is a specialized structure situated between the seminiferous tubule lumen and the capillary network. It is composed of the capillary endothelium, basement membrane, connective tissue, peritubular cells, the seminiferous epithelium basement membrane, and the tight junctions between Sertoli cells [8]. Among these components, the Sertoli cell tight junctions constitute the most critical element of the BTB [9]. Beyond its role in selectively blocking substance passage, the BTB is essential for maintaining a stable microenvironment conducive to the normal proliferation and differentiation of spermatogenic cells [10]. These spermatogenic cells develop under the support of Sertoli cells, which are increasingly recognized not only for providing nourishment but also for actively regulating spermatogenesis [11]. Despite its crucial functions, how the BTB is affected in the context of cryptorchidism remains poorly reported.

In this study, we established a surgical unilateral cryptorchidism model in rats (SUCMR) to comprehensively investigate the modeling process and the spontaneous testicular recovery pattern following orchiopexy. A particular focus was placed on elucidating the dynamic changes in the BTB within this model.

## Methods

### Animals and experimental groups

**Fig. 1:**
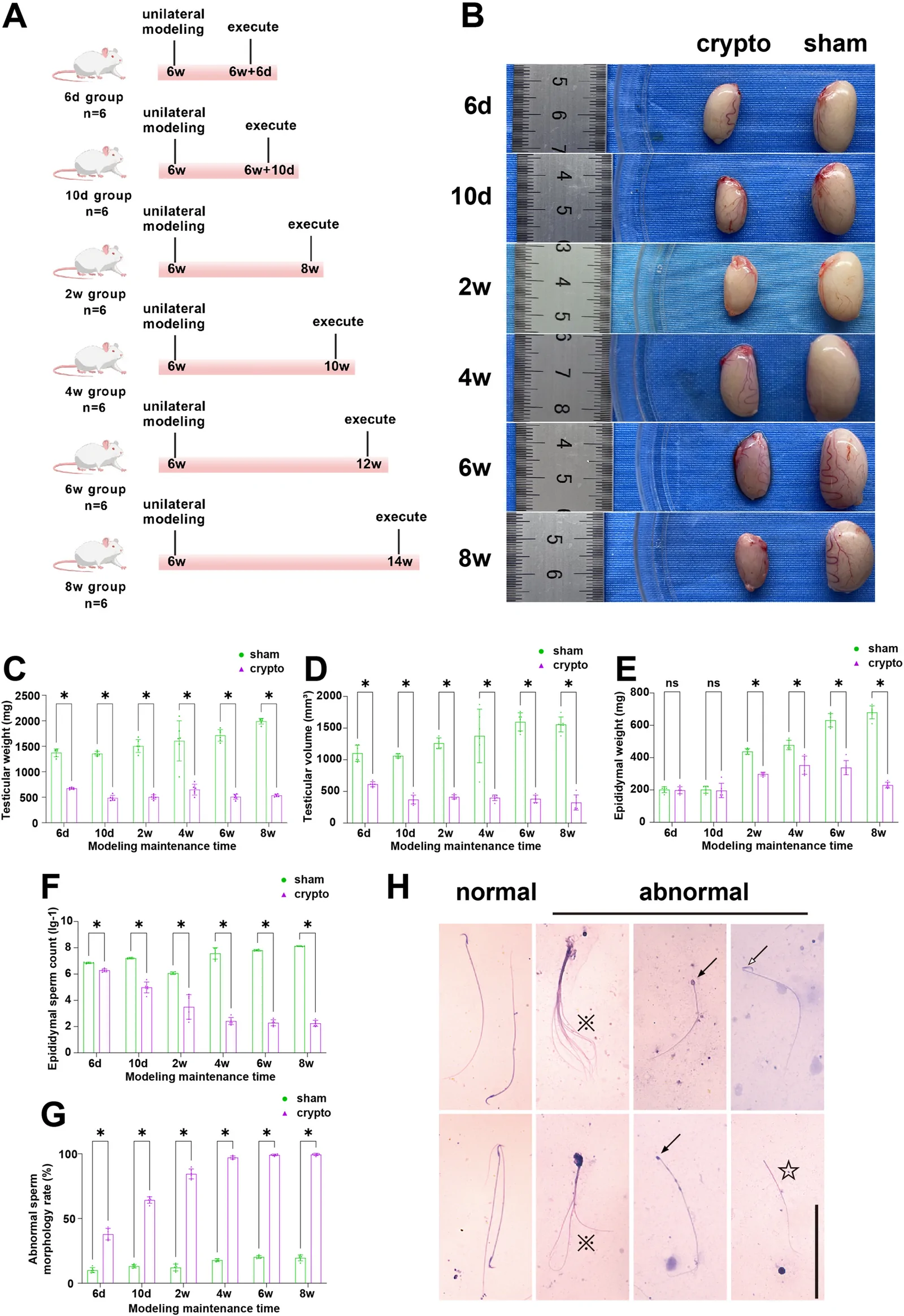
Testicular atrophy and decreased spermatogenic function were induced by the SUCMR. **A** Schematic diagram of animal grouping in the first part of the experiment. **B** Testis appearance of each group in SUCMR. **C**-**G** Testis mass, testis volume, epididymal mass, epididymal sperm count, and sperm malformation rate were statistically analyzed in sham-operated (sham) and cryptorchid (crypto) testes at various time points post-modeling (n = 6). Data were expressed as the mean ± SD, *p < 0.05, significant difference. ns: not significant. **H** Papanicolaou staining for normal sperm and typical sperm deformities. The asterisks showed malformed sperm with multiple tails. The black arrows showed sperm with head deformities. The white arrows showed sperm with detached heads. The pentagrams showed headless sperm. Scale bar = 100 μm

Sprague-Dawley (SD) rats were purchased from SiPeiFu (Beijing, China). The animals were housed individually under controlled conditions (12 h light/12 h dark cycles) with free access to food and water. All experimental protocols were approved by the local ethics committee and conducted in accordance with the guidelines of the Research Ethics Committee of the Second Hospital of Hebei Medical University (Approval No. 2024-AE001). Six-week-old male rats were subjected to unilateral surgical cryptorchidism and subsequently randomized into six groups (*n* = 6). To investigate the effect of cryptorchidism duration on the testis and spermatogenesis, each group of rats was maintained in a cryptorchid state for varying periods: 6 days, 10 days, 2 weeks, 4 weeks, 6 weeks, and 8 weeks (Fig. 1A). To evaluate the potential for functional recovery in the SUCMR, orchiopexy was performed at 6 days, 2 weeks, 4 weeks and 6 weeks after modeling, and spermatogenic function was assessed in 3 and 7 weeks after orchiopexy (Fig. 4A). For euthanasia, rats were euthanized by cervical dislocation.

### Modeling and orchidopexy

SUCMR was established based on previously described methods with modifications [7, 12]. Briefly, rats were anesthetized via an intraperitoneal injection of pentobarbital sodium (25 mg/kg). Hair over the abdomen was removed using a depilatory cream, and a 1.5 cm midline incision was made on the lower abdomen. The right testis was selected for cryptorchidism modeling. The right scrotum was gently pushed to guide the testis into the abdominal cavity, and the gubernaculum of the right testis was then severed. To simulate cryptorchidism, the right testis was secured to the abdominal wall musculature using a *4* − *0* silk suture passed through the tunica albuginea of the upper pole. For the sham-operated side, the left testis underwent identical procedures, including gubernaculum transection and tunica albuginea suturing, but without fixation to the abdominal wall.

For orchidopexy, the procedure was generally similar to clinical orchiopexy. Hair over the abdomen and scrotum was removed using a depilatory cream. A lower abdominal incision, approximately 1.5 cm in length, was made again along the incision made during modeling. The suture knot fixing the right testis to the abdominal wall muscle was cut, and the right testis was fully freed. A longitudinal incision, approximately 3 mm in length, was made in the right scrotum. Ophthalmic forceps were used to probe from the scrotal incision into the abdominal cavity, clamping the lower pole of the right testis and dragging it into the right scrotum. To ensure that the right testis remained in the scrotum, the lower pole of the right testis and the bottom of the right scrotum were sutured together using *4 − 0* silk suture. For the sham-operated side, a scrotal incision was made, the suture knot on the tunica albuginea was removed, and the lower pole of the left testis was also fixed to the left scrotum.

After the main operations of modeling or orchidopexy, bleeding in the abdominal cavity was cleared, and penicillin was injected into the abdominal cavity to prevent infection. Finally, the incisions were sutured in layers using *4 − 0* silk suture. The scrotal muscle was closed with a figure-of-eight suture, the abdominal muscle with continuous sutures, and the scrotal and abdominal skin with interrupted sutures. No further antibiotics were administered postoperatively.

### Epididymal sperm count and papanicolaou staining

The epididymal sperm count was assessed according to a previously described method with minor modifications [13]. After anesthesia, the intact epididymal tissue was isolated and placed in 2 ml of PBS. The tissue was minced with ophthalmic scissors for 1 min into approximately 1 mm³ fragments. The suspension was then incubated in a 37 °C water bath for 30 min to allow sperm release into the medium. After gentle mixing, the supernatant was collected, and sperm numbers were counted using a hemocytometer.

For morphological evaluation, 10 µl of the sperm suspension was smeared onto glass slides, air-dried, and stained using a Fast Papanicolaou Staining Kit (#G1617, Solarbio, Beijing). Briefly, smears were fixed in 95% ethanol for 2 min, rinsed with distilled water for 30 s, stained with nuclear staining solution for 2 min, and rinsed again with tap water. This was followed by staining with cytoplasm staining solution for 2 min. Slides were then rinsed in a decolorization solution for 10 s until no excess dye was observed, air-dried, and mounted with resinene.

### Hematoxylin and Eosin (HE) staining

Testicular tissues were fixed in 4% paraformaldehyde overnight, embedded in paraffin after dehydration and transparency, and sectioned at a thickness of 5 μm. HE staining was performed following standard protocols [14]. Briefly, after deparaffinization and rehydration, sections were stained with hematoxylin solution (#BA4041, BASO, Zhuhai, China) for 5 min, differentiated in 1% acid ethanol for 5 s, and rinsed in distilled water. Subsequently, sections were counterstained with eosin solution (Huntz Bio-Tech, Wuhan, China) for 1 min, dehydrated through a graded ethanol series, and cleared with an Environmentally Friendly Transparent Agent (Huntz Bio-Tech, Wuhan, China). Stained sections were examined and imaged using a LEICA DM6000 microscope (Leica Microsystems, Germany).

### Seminiferous tubule assessment and Johnsen score

To evaluate spermatogenesis, the diameter and the thickness of the seminiferous epithelium were measured, and seminiferous tubules were scored by means of the Johnsen score [15, 16]. For each testis, measurements were performed on 10 approximately circular seminiferous tubules randomly selected from various fields of view. Seminiferous tubules were assigned Johnsen scores on a scale of 1–10: 10: complete spermatogenesis and perfect tubules; 9: many spermatozoa present but disorganized spermatogenesis; 8: only a few spermatozoa present; 7: no spermatozoa but many spermatids present; 6: only a few spermatids present; 5: no spermatozoa or spermatids present but many spermatocytes present; 4: only a few spermatocytes present; 3: only spermatogonia present; 2: no germ cells present and 1: neither germ cells nor Sertoli cells present.

### Biotin tracer assay

The permeability of the BTB was evaluated using a biotin tracer assay as previously described [17]. Anesthetized rats (25 mg/kg pentobarbital sodium) received an intratesticular injection of approximately 100 µl of 10 mg/ml EZ-link Sulfo-NHS-LC-Biotin (#21335, Thermo Scientific, Rockford, IL) beneath the testicular capsule using a 30-gauge needle. After 40 min, the rats were euthanized, and the testes were immediately harvested and fixed in 4% paraformaldehyde. Paraffin sections were incubated with Alexa Fluor™ 488-conjugated streptavidin (#S11223, Thermo Scientific, Rockford, IL) at 37 °C for 1 h. Nuclei were counterstained with 4′,6-diamidino-2-phenylindole (DAPI) for 10 min. Slides were mounted with neutral balsam and visualized under a LEICA DM6000 microscope (Leica Microsystems, Germany).

### Transmission Electron Microscopy (TEM)

Testicular samples for TEM were prepared as described previously [18]. Briefly, testes were dissected into 1 mm³ cubes and fixed in 4% glutaraldehyde (#P1127, Solarbio, Beijing), followed by embedding in Araldite resin. Ultrathin Sect. (50 nm) were stained with 1% uranyl acetate and Reynold’s lead citrate and examined using a Hitachi H-7650 transmission electron microscope (Hitachi, Tokyo, Japan).

### Western blot analysis

The following primary antibodies were used: anti-ZO-1 (#21773-1-AP), anti-OCCLUDIN (#66378-1-Ig), anti-β-ACTIN (#66009-1-Ig), anti-rabbit IgG-HRP (#RGAR001), and anti-mouse IgG-HRP (#RGARM001) from Proteintech (Wuhan, China); anti-CLAUDIN-11 (#36-4500) from Thermo Scientific (Rockford, IL, USA).

Testis tissues were homogenized in RIPA lysis buffer (#32010, Bestbio, China) supplemented with a protease inhibitor cocktail. Protein extracts were separated by 10% or 12% SDS-polyacrylamide gel electrophoresis and transferred to PVDF membranes (#SW221-02, Seven, China). The membranes were probed with specific primary antibodies, followed by incubation with appropriate horseradish peroxidase (HRP)-conjugated secondary antibodies. Protein bands were detected using a chemiluminescent HRP substrate (#BMU102, Abbkine, China). β-ACTIN was used as a loading control for normalization.

### Statistical analysis

Data are presented as the mean ± standard error of the mean (SEM) from at least three independent experiments. The normality of data distribution was assessed using the Shapiro-Wilk test, and homogeneity of variances was verified using Levene’s test. Comparisons between the sham-operated and model sides within the same animal were analyzed using a paired t-test or the Wilcoxon signed-rank test. For comparisons between two independent groups, an unpaired t-test or the Mann-Whitney U test was applied. Multiple group comparisons were performed using one-way analysis of variance (ANOVA) or the Kruskal-Wallis test. A p-value of less than 0.05 was considered statistically significant.

## Results

### SUCMR Induces seminiferous epithelium sloughing, leading to testicular atrophy and spermatogenic impairment

Compared to the sham-operated (sham) testes, the cryptorchid (crypto) testes exhibited significant atrophy (Fig. 1B). Even after only 6 days of modeling, significant reductions in testis mass and volume were observed (Fig. 1CD), and testicular atrophy progressively worsened with the extension of the modeling period. As an important site for sperm maturation, the epididymal mass was also statistically analyzed. During the initial phase of the SUCMR, the reduction in epididymal mass was not pronounced; however, by 2 weeks of modeling, a significant decrease in epididymal mass on the crypto side was observed, which further diminished at 8 weeks of modeling (Fig. 1E). In contrast to the changes observed in epididymal mass, SUCMR exerted a more rapid effect on epididymal sperm count and sperm malformation rate. By day 6 of modeling, a significant reduction in epididymal sperm count was already evident on the crypto side (Fig. 1F), accompanied by a marked increase in the sperm malformation rate (Fig. 1G). As the modeling period progressed, the sperm count continued to decline sharply (Fig. 1F), and the sperm malformation rate escalated, nearly reaching 100% at 6 and 8 weeks post-modeling (Fig. 1G). Among the malformed spermatozoa, the most frequently observed abnormalities included multiple tails, head deformities, detached heads, and headless sperm (Fig. 1H). Among spermatozoa with multiple tails, the majority possessed 2–4 tails, although a small number of malformed spermatozoa were noted to have as many as 7–8 tails (Fig. 1H).

**Fig. 2:**
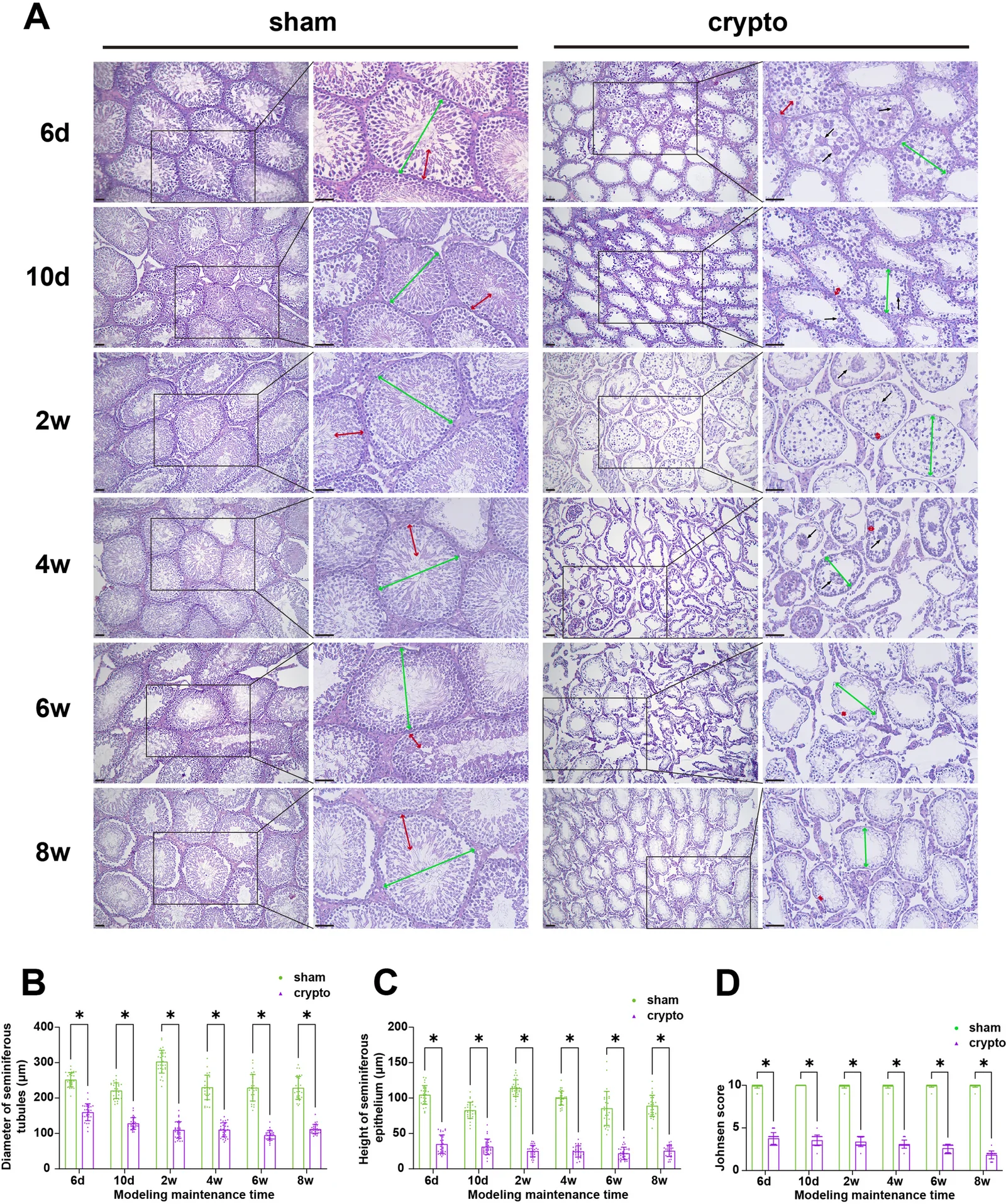
Seminiferous tubule atrophy and seminiferous epithelium exfoliation were observed in the SUCMR. **A** Paraffin sections of testes from sham and crypto sides at various time points post-modeling were subjected to HE staining. The seminiferous tubule diameter was indicated by green double arrows, the spermatogenic epithelium thickness was indicated by red double arrows, and sloughed seminiferous epithelium was indicated by black arrows. Scale bar = 100 μm. **B**-**C** Seminiferous tubule diameter, seminiferous epithelium thickness and Johnsen score were statistically analyzed in sham and crypto testes at various time points post-modeling (n = 3). For each testis, ten seminiferous tubules with near-circular profiles were randomly selected and counted. Data were expressed as the mean ± SD, *p < 0.05, significant difference

HE staining of testicular tissues from all groups revealed a significant collapse of seminiferous tubules in the crypto testes. Within these tubules, sperm cells were almost entirely absent, with only early germ cells occasionally observed, and numerous clumps of desquamated seminiferous epithelium were observed (Fig. 2A). This shedding of seminiferous epithelium was persistent, with desquamated epithelial cells consistently observed within the seminiferous tubules throughout the first 4 weeks of the modeling period. By the 8th week of modeling, the seminiferous tubules exhibited complete cavitation, but Sertoli cells persisted (Fig. 2A). To further quantify these changes in the seminiferous tubules, their diameter and the thickness of the seminiferous epithelium and Johnsen score were measured. The results indicated a progressive decrease in seminiferous tubule diameter (Fig. 2B), seminiferous epithelium thickness (Fig. 2C) and Johnsen score (Fig. 2D) with increasing modeling duration, reaching a stable low level by weeks 6 and 8.

In conclusion, SUCMR can induce the progressive sloughing of the seminiferous epithelium, resulting in testicular atrophy and impaired spermatogenesis. This reproductive damage was observed to worsen gradually with extended modeling time.

### SUCMR disrupts the blood-testis barrier

**Fig. 3:**
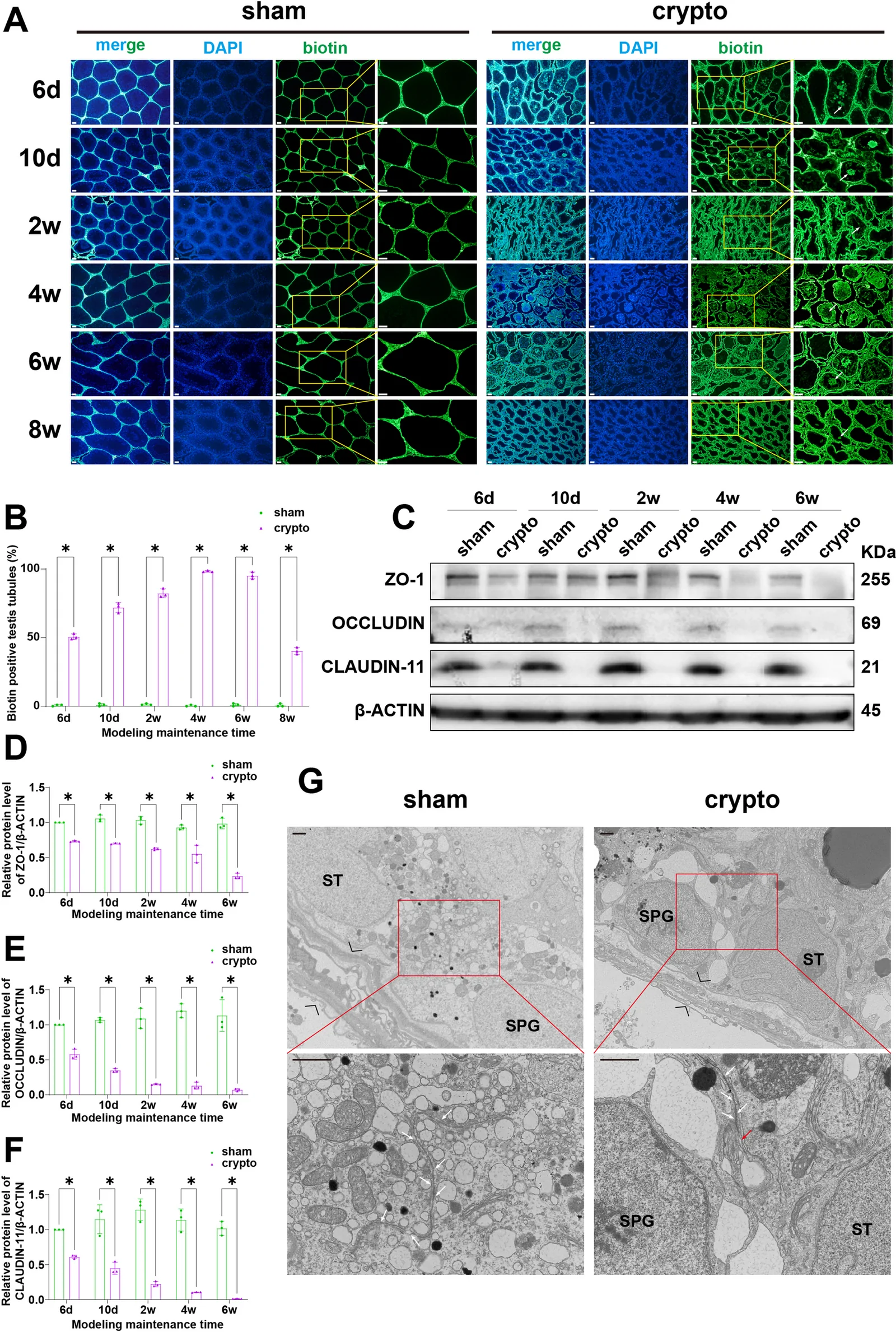
Blood-testis barrier injury was caused by SUCMR. **A** Biotin tracing staining was performed on sham and crypto testes at various modeling time points. White arrows indicated distinctly stained seminiferous epithelium. Scale bar = 100 μm. **B** The ratio of biotin-positive seminiferous tubules in sham and crypto sides was statistically analyzed at different modeling time points (n = 3). For each testis, 100 seminiferous tubules were counted in random fields of view. Data were expressed as the mean ± SD, *p < 0.05, significant difference. **C** The expression of blood-testis barrier marker proteins ZO-1, OCCLUDIN, and CLAUDIN-11 was detected in the sham and crypto testes at different modeling time points via Western blot analysis. **D**-**F** Quantification of the relative expression levels of ZO-1, OCCLUDIN, and CLAUDIN-11 in panel C from three independent rats. Data were expressed as the mean ± SD, *p < 0.05, significant difference. **G** Transmission electron microscopy images of testicular tissues at 2 weeks after the establishment of the SUCMR. ST: Sertoli cell nucleus, SPG: spermatogonial nucleus. The large arrow showed the basement membrane of the seminiferous tubules. The small white arrow showed normal tight junction structures. The small red arrow showed broken tight junction structures. Scale bar = 1 μm

The BTB provides a stable developmental microenvironment for the seminiferous epithelium, with Sertoli cells surrounding and anchoring germ cells at various stages within the seminiferous tubules [19]. We hypothesized that the shedding of the seminiferous epithelium would be accompanied by injury to the BTB. To verify this hypothesis, biotin tracing experiments were performed on the testes in each group. Consistent with our hypothesis, significant biotin leakage was observed within the seminiferous tubules on the crypto side, while almost no biotin was detected within the seminiferous tubules on the sham side (Fig. 3A). To quantify the biotin tracing experiment, 100 seminiferous tubules were counted in random fields of each testis, and the biotin positive rate was subsequently calculated (*n* = 3). Results indicated that by 6 days after modeling, the crypto testes exhibited significant biotin leakage. As the modeling duration was extended, the biotin positive rate within the seminiferous tubules of the crypto side progressively increased (Fig. 3B). However, by 8 weeks following modeling, the biotin positive rate in the crypto seminiferous tubules showed a subsequent decrease compared to earlier observations. This reduction was attributed to the almost complete cavitation of the seminiferous tubules with only Sertoli cells remaining, meaning that even if biotin extravasated, there were no cellular structures available for its attachment.

Subsequently, western blot (WB) experiments were conducted to examine the expression of key BTB proteins, namely ZO-1, OCCLUDIN, and CLAUDIN-11 [20]. It was found that all three proteins were significantly reduced in the crypto testis (Fig. 3C), and quantitative analysis showed that their expression levels gradually declined as the modeling period lengthened (Fig. 3D-F). Transmission electron microscopy images of testicular tissue from both the sham side and the crypto side at 2 weeks post-modeling revealed breaks in tight junctions (Fig. 3G).

In this part of the research, it was demonstrated that the SUCMR impairs the integrity of the BTB and leads to a progressive decrease in the expression of the key barrier proteins ZO-1, OCCLUDIN, and CLAUDIN-11.

### Testicular atrophy and spermatogenic impairment induced by short-term SUCMR are reversible

**Fig. 4:**
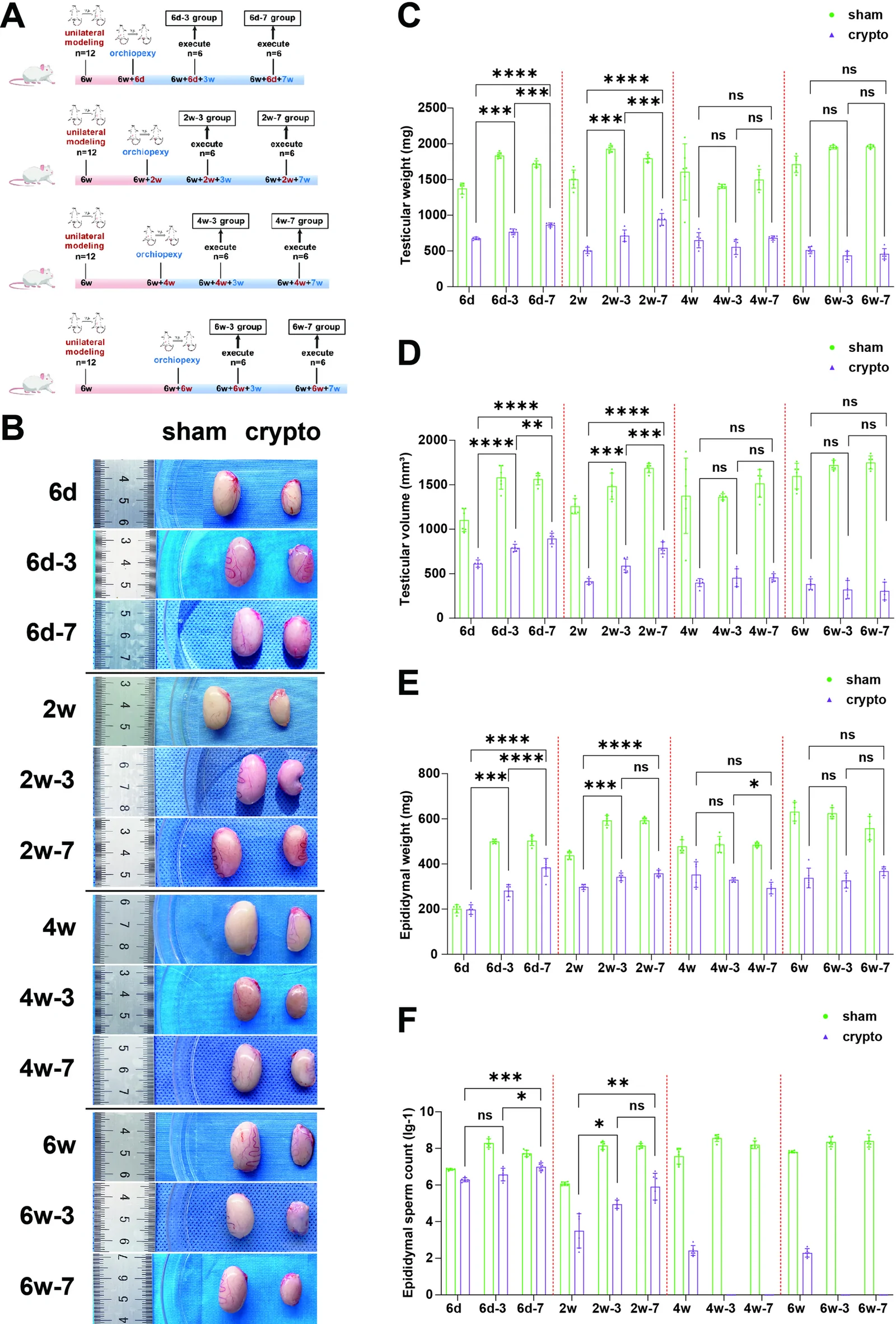
Testicular atrophy and spermatogenic dysfunction induced by short-term model were reversible. **A** Schematic diagram of animal grouping in the second part of the experiment. **B** Appearance of sham and crypto testes in each group of rats following orchiopexy at different time points in the SUCMR. **C**-**F** Testis mass, testis volume, epididymal mass, and epididymal sperm count were statistically analyzed in each group of rats following orchiopexy at different time points in the SUCMR (*n* = 6). Data were expressed as the mean ± SD, *p < 0.05, **p < 0.01, ***p < 0.001, ****p < 0.0001, significant difference. ns: not significant

Timely orchiopexy represents the primary clinical intervention for pediatric cryptorchidism, with the goal of restoring spermatogenic function [21]. To evaluate the potential for functional recovery in the SUCMR, orchiopexy was performed at different time points after modeling, and spermatogenic function was assessed during the recovery phase (Fig. 4A). Gross examination indicated that the cryptorchid testes remained noticeably smaller than those on the sham side. However, in rats that had undergone cryptorchidism for up to 2 weeks, the cryptorchid testes showed considerable improvement in appearance after being returned to the scrotum (Fig. 4B). Quantitative results confirmed that testicular mass and volume were significantly restored in groups that underwent orchiopexy after 6 days and 2 weeks of modeling (Fig. 4CD). In contrast, when the modeling duration was extended to 4 or 6 weeks, orchiopexy failed to produce significant improvement in testicular mass or volume; instead, a trend toward further deterioration was observed (Fig. 4CD).

A similar pattern was observed for epididymal mass and sperm count. In rats with modeling durations within 2 weeks, orchiopexy facilitated a recovery in epididymal mass and sperm count (Fig. 4EF). However, in the 4-week and 6-week model groups, no significant recovery in either epididymal mass or sperm count was observed following orchiopexy (Fig. 4EF).

**Fig. 5:**
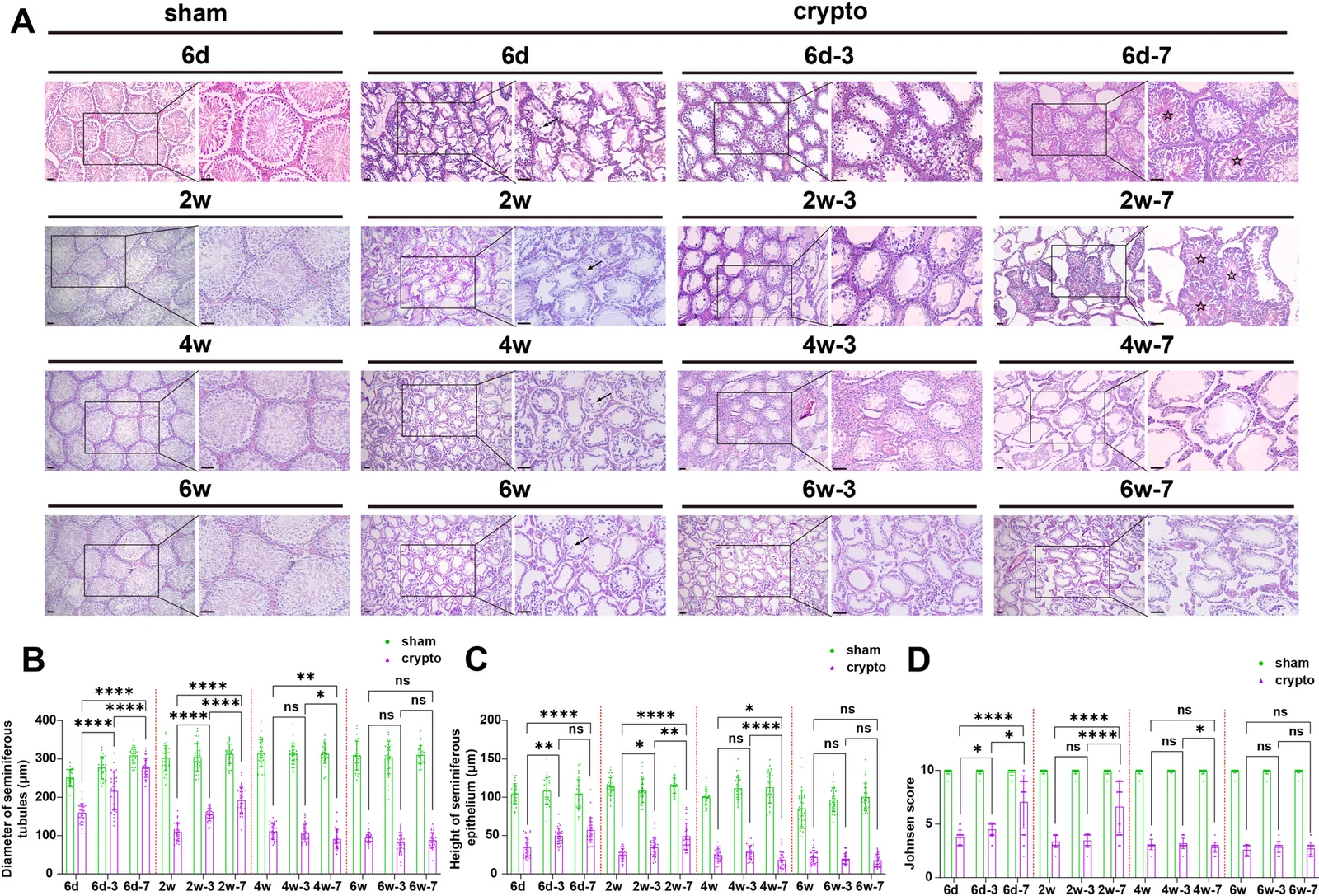
Pathological manifestations were irreversible in SUCMR maintained for 4 weeks or longer. **A** In SUCMR, sham and crypto testes from each group were subjected to HE staining of paraffin sections at various time points following orchiopexy. Seminiferous tubules exhibiting restored seminiferous epithelium were indicated by asterisks. Scale bar = 100 μm. **B**-**D** Following orchiopexy at different time points in SUCMR, seminiferous tubule diameter, seminiferous epithelium thickness and Johnsen score were statistically analyzed (n = 3). For each testis, ten seminiferous tubules with near-circular profiles were randomly selected and counted. Data were expressed as the mean ± SD, *p < 0.05, **p < 0.01, ***p < 0.001, ****p < 0.0001, significant difference. ns: not significant.

Furthermore, HE staining was performed on the descended testes at different time points after SUCMR. In both the 6d-7 and 2w-7 groups, seminiferous tubules with intact spermatogenic epithelium were observed in the cryptorchid testes, including early, mid, and late-stage spermatogenic cells and mature spermatozoa. The recovery of the seminiferous tubules was not uniform, and cavitated tubules were still present. Notably, the number of cavitated seminiferous tubules was lower in the 6d-7 group compared to the 2w-7 group (Fig. 5A). For each testis, 10 approximately circular seminiferous tubules were randomly selected for measurement of seminiferous tubule diameter, spermatogenic epithelium thickness and Johnsen score (*n* = 3). The results confirmed that orchiopexy performed within 2 weeks of SUCMR enabled significant recovery in seminiferous tubule diameter, epithelial thickness and Johnsen score, whereas this recovery trend was absent in rats subjected to 4 or 6 weeks of modeling (Fig. 5BCD).

### Blood-testis barrier disruption becomes irreversible after 4 weeks of sustained SUCMR

**Fig. 6:**
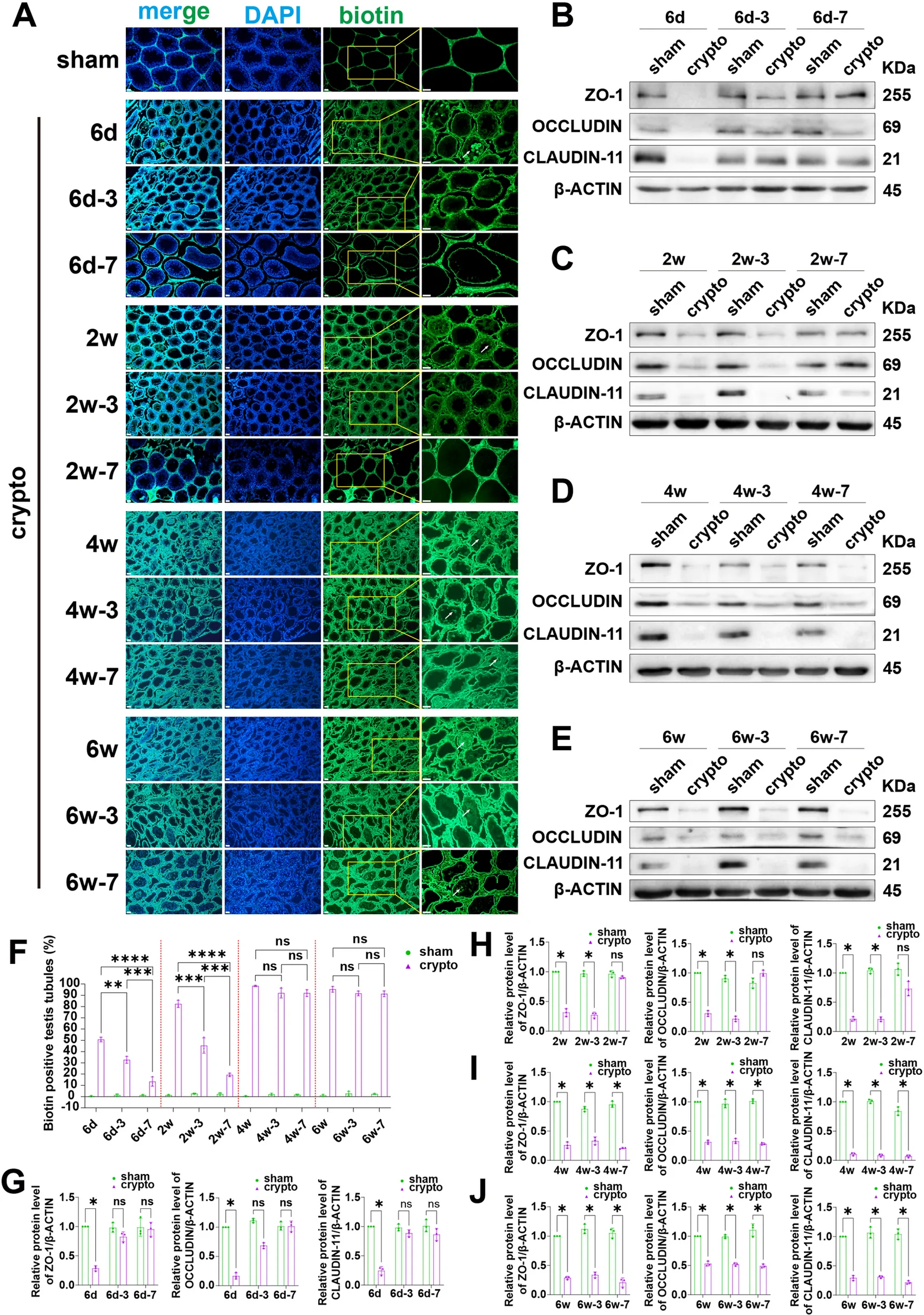
Blood-testis barrier injury was irreversible in SUCMR maintained for 4 weeks or longer. **A** Following orchiopexy at different time points in SUCMR, biotin tracing staining was performed on sham and crypto testes. White arrows indicated distinctly stained seminiferous epithelium. Scale bar = 100 μm. **B**-**E **Following orchiopexy at different time points in SUCMR, the expression of ZO-1, OCCLUDIN, and CLAUDIN-11 was detected in the sham and crypto testes. **F** Following orchiopexy at different time points in SUCMR, the ratio of biotin-positive seminiferous tubules in sham and crypto sides was statistically analyzed (n = 3). For each testis, 100 seminiferous tubules were counted in random fields of view. Data were expressed as the mean ± SD, **p < 0.01, ***p < 0.001, ****p < 0.0001, significant difference. ns: not significant. **G**-**J** Quantification of relative expression of protein ZO-1、OCCLUDIN、and CLAUDIN-11 in (**B**-**E**) of three independent rats. Data were expressed as the mean ± SD, *p < 0.05, significant difference. ns: not significant.

To determine whether BTB integrity in cryptorchid testes could recover following orchiopexy, biotin tracing assays were conducted on testes from each group. BTB integrity showed significant improvement in descended testes that had been subjected to SUCMR for 6 days or 2 weeks (Fig. 6A). However, in testes that had undergone SUCMR for 4 weeks or 6 weeks, BTB integrity failed to recover within the 7-week observation period after orchiopexy (Fig. 6A). A consistent pattern was observed in the ratio of biotin-positive seminiferous tubules (*n* = 3) (Fig. 6F).

We further employed western blotting to examine the expression levels of three key BTB proteins: ZO-1, OCCLUDIN, and CLAUDIN-11 (Fig. 6B-E). Quantitative analysis demonstrated that the expression of these three proteins was significantly higher in descended testes from the 6-day and 2-week SUCMR groups following orchiopexy (Fig. 6GH). In contrast, for testes subjected to 4 weeks or 6 weeks of SUCMR, the protein levels of ZO-1, OCCLUDIN, and CLAUDIN-11 showed no significant recovery within 7 weeks after orchiopexy (Fig. 6IJ).

## Discussion

In this study, we used a surgical unilateral cryptorchidism model in 6-week-old rats to characterize time-dependent changes in spermatogenic function and blood-testis barrier (BTB) integrity, as well as their recovery after the testis was returned to the scrotum. This model provides a controlled experimental system in which the onset and duration of postnatal abdominal testicular exposure can be standardized. Importantly, however, it does not reproduce the developmental history of congenital human cryptorchidism. The testes in our rats had already descended and developed in the scrotum before being surgically relocated to the abdomen, whereas human cryptorchidism originates during abnormal testicular descent and may involve prenatal endocrine, genetic, and anatomical factors. Therefore, the present findings should be interpreted primarily as model-specific observations of testicular injury and recovery after experimentally induced postnatal abdominal relocation, rather than as a direct representation of the natural history or treatment response of human cryptorchidism.

Currently, early surgical intervention is the primary treatment for cryptorchidism [22]. However, orchiectomy is sometimes necessary in cases of severe testicular atrophy to mitigate the risk of carcinoma in adulthood [23]. Despite this, for various reasons, many patients or their guardians choose orchiopexy alone, thereby retaining severely atrophied testicular tissue. Moreover, due to the functional importance of testicular tissue, intraoperative biopsy of even mildly atrophied testes raises ethical concerns [24]. Consequently, obtaining sufficient human cryptorchid tissue samples for clinical research remains challenging. A stable and reliable animal model is therefore essential for advancing the study of this condition. In our study, a surgical model was used to simulate cryptorchidism. Based on the rats’ tolerance to surgical injury and previous studies [7, 12], we selected 6 weeks of age as the modeling time point [7, 12], we selected six weeks after birth as the modeling time point. Besides surgical modeling, previous studies have employed other methods to induce cryptorchidism, including prenatal administration of anti-androgens such as flutamide [25] and dibutyl phthalate [26], as well as postnatal subcutaneous hormone injections [27]. Cryptorchidism phenotypes have also been reported in transgenic mice overexpressing aromatase (CYP19A1) [28] and insulin-like 3 (INSL-3) [29]. Furthermore, congenital models such as Long-Evans/Cryptorchid (LE/Orl) rats [30] and trans-scrotal (TS) rats [31] are available. However, the use of pharmacological agents, genetic modifications, or specific rat strains can introduce confounding physiological disturbances beyond cryptorchidism itself. These additional variables, often difficult to fully characterize, may limit the model’s fidelity to the human disease. In contrast, surgical models allow relatively precise control over the onset and duration of abdominal testicular exposure and reduce some systemic confounding effects. These experimental advantages, however, do not imply that surgical models reproduce the full developmental and etiological complexity of human cryptorchidism. We therefore selected the SUCMR to investigate selected local testicular responses to controlled postnatal abdominal exposure and subsequent return to the scrotum, rather than to model the entire human disease process.

Although earlier orchiopexy is generally associated with better preservation of testicular function in children with cryptorchidism [32], direct temporal comparison between clinical cryptorchidism and the present rat model is not appropriate. In the SUCMR, testicular injury became progressively more severe with longer abdominal retention. After shorter modeling durations, orchiopexy was followed by recovery of several measured parameters. However, histological recovery was heterogeneous, and cavitated seminiferous tubules remained in some testes. In contrast, after 4 or 6 weeks of modeling, no significant recovery was detected during the 7-week post-orchiopexy observation period. These findings support a duration-dependent and time-sensitive pattern of injury and recovery within this experimental system. Importantly, the 2-week and 4-week intervals identified here are specific to the age of the rats, the experimental procedure, and the duration of follow-up, and should not be interpreted as equivalents of clinical treatment windows in humans. For future experiments using the same SUCMR protocol, a modeling duration of at least 4 weeks may provide a relatively stable injury state with less spontaneous recovery.

The etiology of human cryptorchidism is multifactorial and may involve fetal hormonal disturbances, abnormal gubernacular development, genetic factors, and local anatomical abnormalities. Elevated temperature associated with an extra scrotal position is considered an important contributor to subsequent testicular injury [33], but it is unlikely to represent the only pathogenic mechanism. Germ cell apoptosis is a primary characteristic of this testicular injury. Previous studies have reported that undescended testes induce testicular oxidative stress [34], which consequently leads to spermatogenic cell death [35]. The SUCMR primarily models the consequences of postnatal abdominal testicular exposure, while bypassing the prenatal and developmental abnormalities involved in congenital human cryptorchidism. In addition, effects related to surgical manipulation and testicular fixation cannot be completely excluded. Within this experimental context, extensive seminiferous epithelial sloughing was accompanied by early disruption of BTB integrity and a progressive reduction in the expression of ZO-1, OCCLUDIN, and CLAUDIN-11. These associations raise the possibility that Sertoli cell and BTB dysfunction may contribute to spermatogenic injury in this model. However, the present study was not designed to establish a causal relationship or to determine whether BTB disruption precedes germ-cell loss.

In previous animal models of cryptorchidism, damage and apoptosis of spermatogenic cells have been reported, as well as disruption of the blood-testis barrier. Ferragut Cardoso AP et al. also investigated the time course in a rat model of cryptorchidism; despite variations in the initial time point for model induction, the general patterns observed were similar [36]. Zeng et al. demonstrated that cryptorchidism disrupts the BTB by activating Sertoli cell ferroptosis through oxidative stress [37]. Sertoli cell dysfunction has also been observed in the cryptorchid testes of yaks [38]. However, many previous reports are single time-point studies, and the temporal patterns of BTB injury have not been explored in detail. There are relatively few studies on tight junctions in human cryptorchidism samples, but research by JC Cavicchia et al. in human cryptorchidism testis samples also supports the existence of BTB damage [39]. The integrity and normal function of the BTB are essential for spermatogenesis [40]. Sertoli cells, the primary nourishing cells, play a critical regulatory role in this process. It has been previously suggested that Sertoli cell tight junctions divide the seminiferous epithelium into two compartments, regulating the proliferation and differentiation of spermatogonia [41]. Beyond tight junctions, intercellular communication also occurs between Sertoli cells and spermatogenic cells at all stages of development [42]. In our study, disruption of BTB integrity was observed in SUCMR during the early stages of modeling, preceding significant spermatogenic cell apoptosis. Furthermore, prolonged modeling time led to a gradual decrease in tight junction protein expression, accompanied by a decline in spermatogenic function, as evidenced by reduced sperm count. Notably, a completely restored blood-testis barrier was observed in rats that underwent orchiopexy early in the modeling process, with a concurrent recovery of spermatogenic function indicators, including sperm count. This indicates that Sertoli cell dysfunction may occur prior to spermatogenic cell apoptosis, and that damage to the BTB is entirely reversible in the early phase of cryptorchidism.

Given the 48-day spermatogenic cycle in rats, the maximum observation period following testicular descent was set at 7 weeks. However, whether the recovery of spermatogenic epithelium after surgical correction of cryptorchidism can be observed over extended spermatogenic cycles remains uncertain. Meanwhile, studies have observed disruption of BTB integrity [43, 44]. In this study, we selected the contralateral testis as a sham-operated control. However, a limitation was the absence of a completely normal control group. Additionally, the relatively short duration of the model prevented a comprehensive evaluation of the long-term effects of unilateral cryptorchidism on the contralateral testis. Given that testicular descent in rats occurs between 21 and 28 days after birth, at 6 weeks of age, the testes of the rats in our model had already developed normally in the scrotum for approximately 2 weeks. This is one of the limitations of our study. In subsequent studies, we will perform modeling at an earlier age to minimize this confounding effect. Furthermore, while surgical models offer improved testicular specificity and minimize systemic effects compared to drug-induced models, extrapolating findings from animal models to human disease is limited by species differences. Additionally, the absence of fertility studies prevents a direct assessment of SUCMR in terms of reproductive capacity.

## Conclusions

This study revealed that SUCMR resulted in reduced testis mass and volume, as well as decreased epididymal mass and epididymal sperm count. Concurrently, an elevated sperm malformation rate, diminished seminiferous tubule diameter and seminiferous epithelium thickness were observed, alongside compromised blood-testis barrier integrity and reduced expression of key tight junction proteins. These alterations were exacerbated with increasing modeling duration, peaking at 6 weeks. Orchiopexy can effectively restore the multifaceted testicular damage induced by SUCMR within a two-week period. However, the damage resulting from a 4-week modeling period could not be reversed by orchiopexy.

## Supplementary Information

Supplementary Material 1.

## Data Availability

The datasets used and/or analysed during the current study are available from the corresponding author on reasonable request.

## Abbreviations

BTB: blood-testis barrier
SUCMR: surgical unilateral cryptorchidism model in rats
SD: Sprague-Dawley
HE: Hematoxylin and Eosin
TEM: Transmission Electron Microscopy
HRP: horseradish peroxidase
