# Supplementary material for: Injury and recovery patterns of the blood-testis barrier and spermatogenic function in a surgical unilateral cryptorchidism model in rats
Source: Reprod Biol Endocrinol. 2026 Jul 16;24:95. doi: 10.1186/s12958-026-01590-5 (PMC13656716; doi:10.1186/s12958-026-01590-5)
Supplement: Supplementary file 1 [file 12958_2026_1590_MOESM1_ESM.pdf]

Figure3C

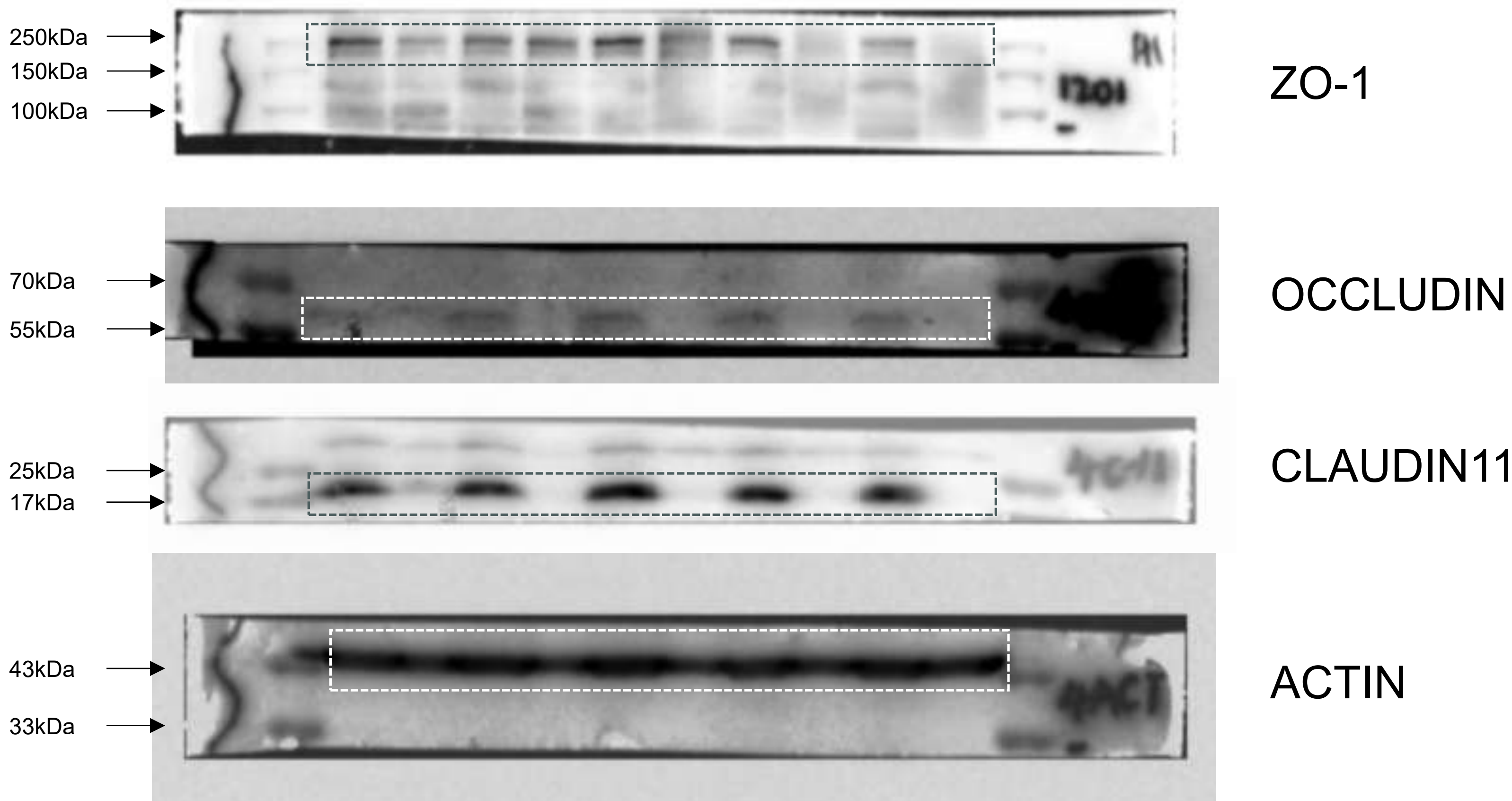
250kDa
150kDa
100kDa
ZO-1
70kDa
55kDa
OCCLUDIN
25kDa
17kDa
CLAUDIN11
43kDa
33kDa
ACTIN

Figure6B

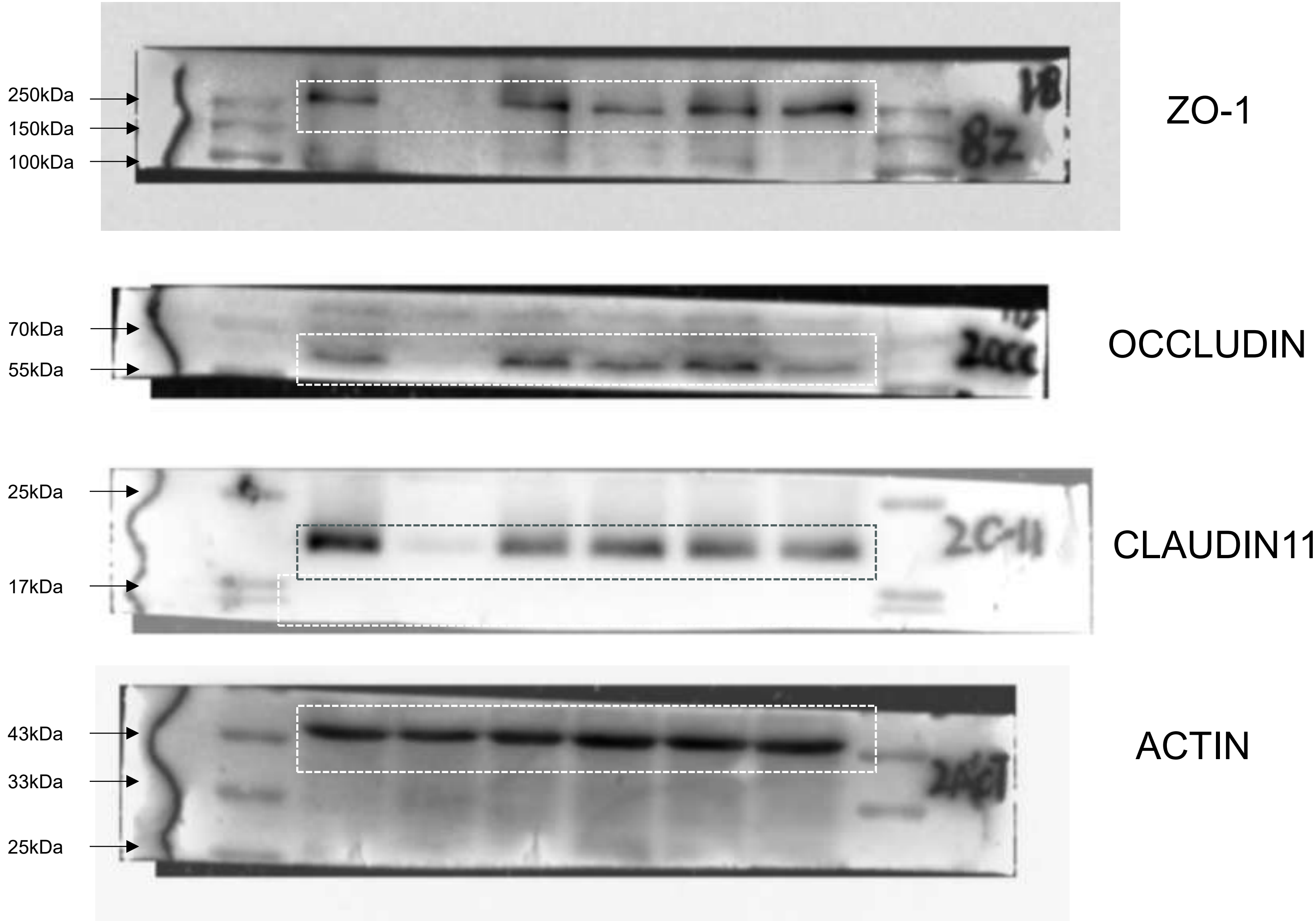
250kDa
150kDa
100kDa
ZO-1
70kDa
55kDa
OCCLUDIN
25kDa
17kDa
CLAUDIN11
43kDa
33kDa
25kDa
ACTIN

Figure6C

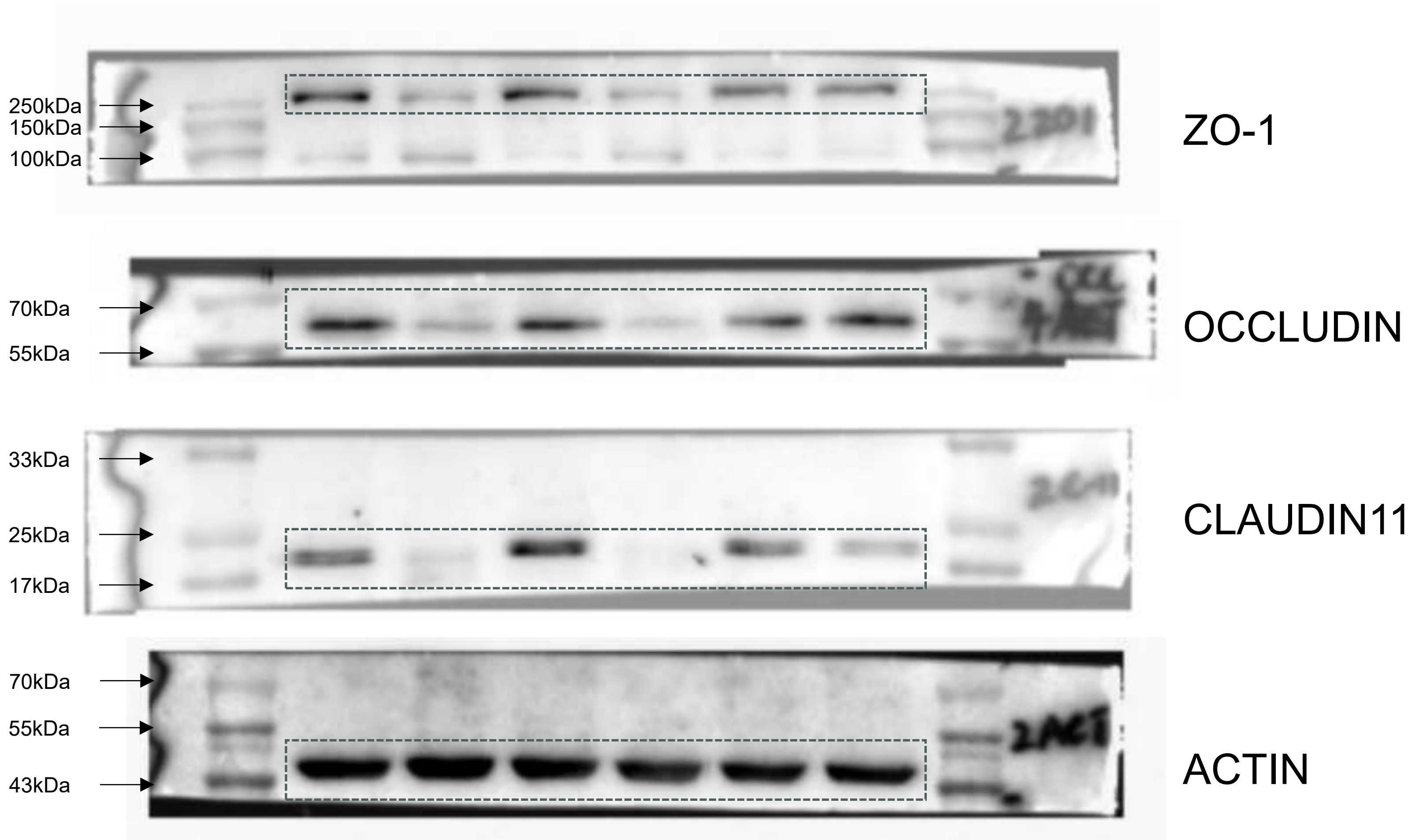
250kDa
150kDa
100kDa
ZO-1
70kDa
55kDa
OCCLUDIN
33kDa
25kDa
17kDa
CLAUDIN11
70kDa
55kDa
43kDa
ACTIN

Figure6D

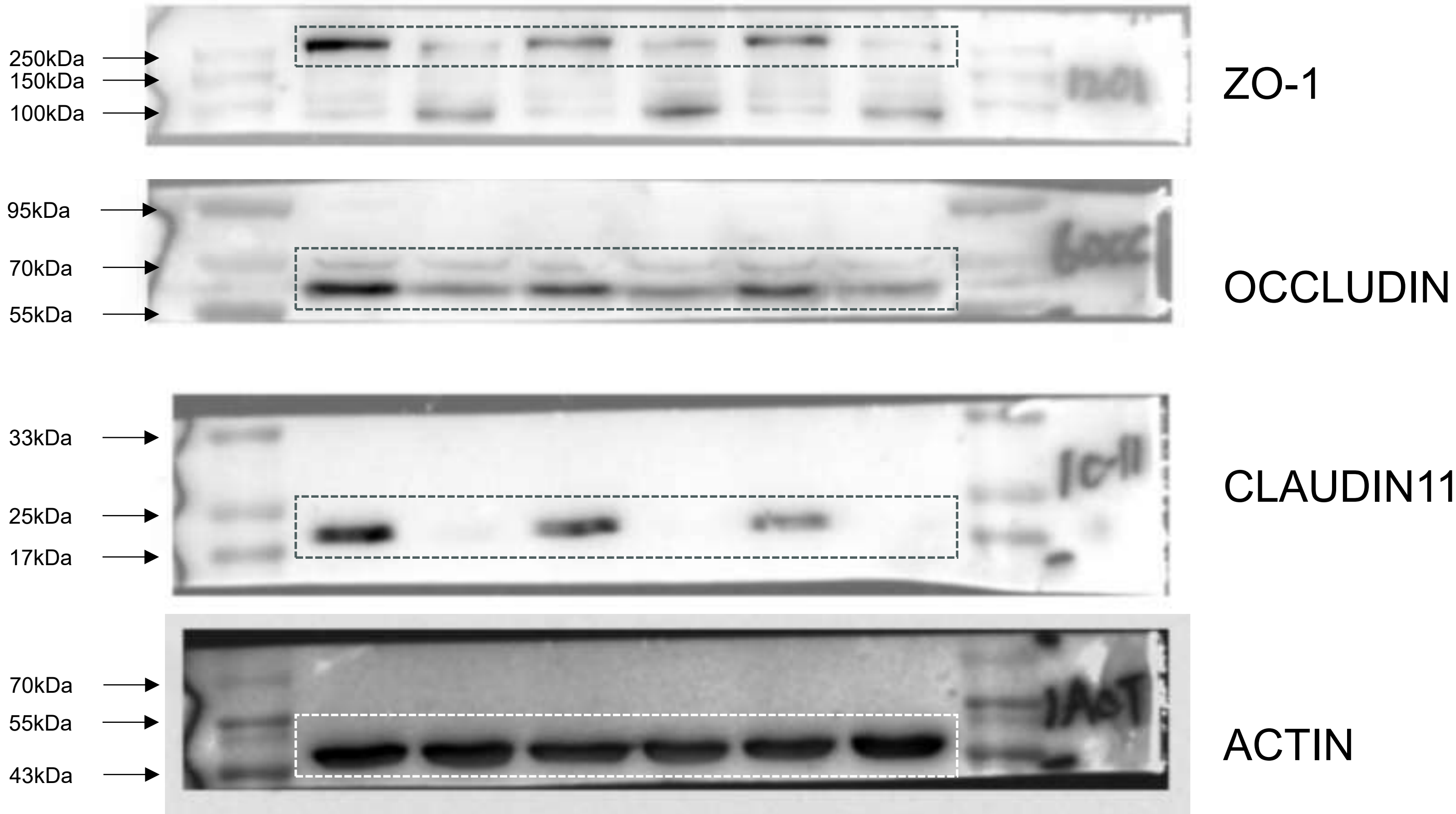
250kDa
150kDa
100kDa
ZO-1
95kDa
70kDa
55kDa
OCCLUDIN
33kDa
25kDa
17kDa
CLAUDIN11
70kDa
55kDa
43kDa
ACTIN

Figure6E

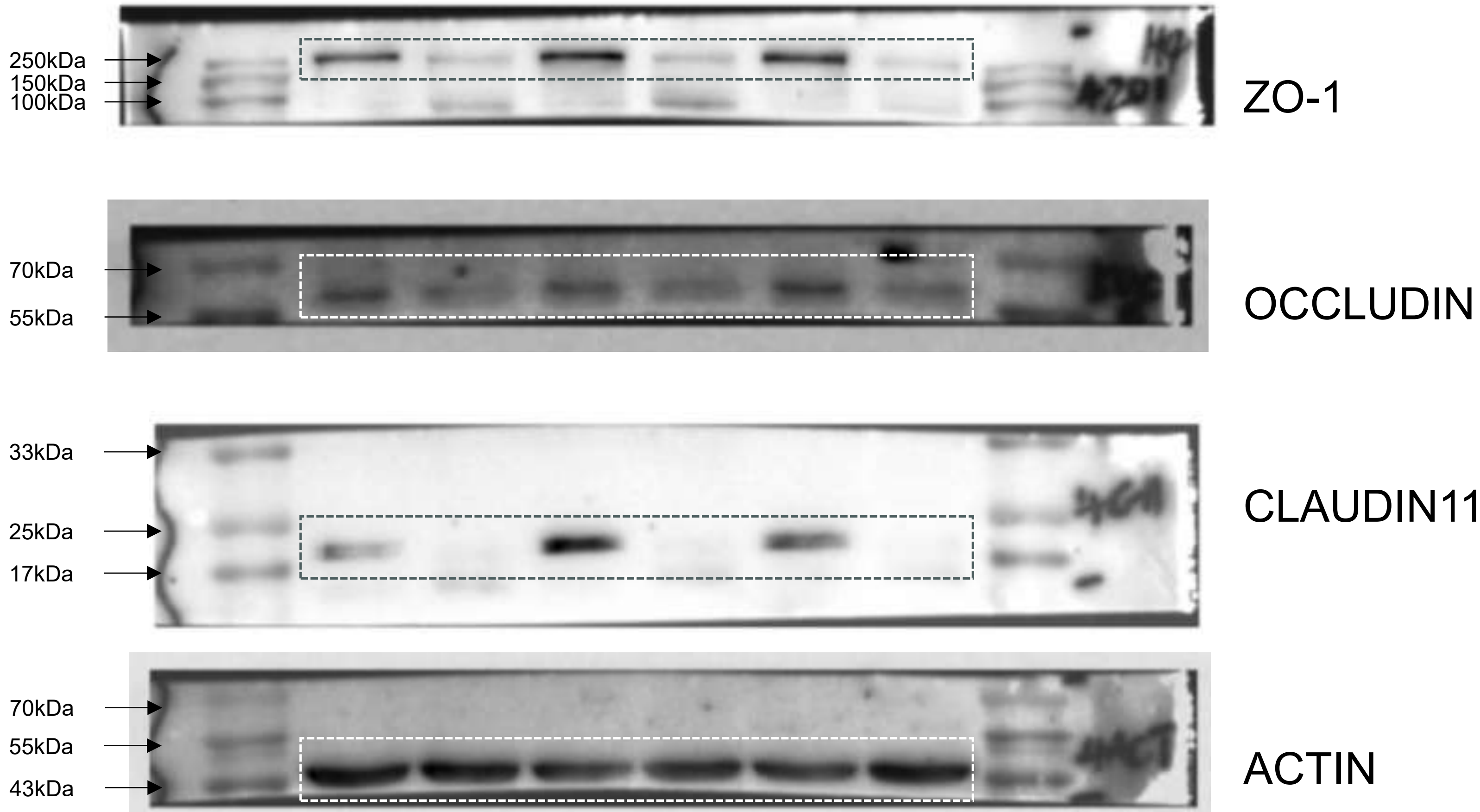
250kDa
150kDa
100kDa
ZO-1
70kDa
55kDa
OCCLUDIN
33kDa
25kDa
17kDa
CLAUDIN11
70kDa
55kDa
43kDa
ACTIN
